# Functionality appreciation is inversely associated with positive psychotic symptoms in overweight/obese patients with schizophrenia

**DOI:** 10.1186/s12888-023-04795-9

**Published:** 2023-05-01

**Authors:** Daniella Mahfoud, Feten Fekih-Romdhane, Jawad Abou Zeid, Lea Rustom, Charbel Mouez, Georges Haddad, Souheil Hallit

**Affiliations:** 1 Research Department, Psychiatric Hospital of the Cross, Jal Eddib, Lebanon; 2 The Tunisian Center of Early Intervention in Psychosis, Department of Psychiatry “Ibn Omrane”, Razi hospital, Manouba, 2010 Tunisia; 3 Faculty of Medicine of Tunis, Tunis El Manar University, Tunis, Tunisia; 4 School of Medicine and Medical Sciences, Holy Spirit University of Kaslik, P.O. Box 446, Jounieh, Lebanon; 5 Applied Science Research Center, Applied Science Private University, Amman, Jordan

**Keywords:** Functionality appreciation, Body appreciation, Weight stigma, Positive psychotic symptoms, Schizophrenia, Obesity

## Abstract

**Background:**

While the relationship between negative aspects of body image and positive schizophrenia symptoms was extensively investigated and is relatively well-established, there is a dearth of literature on the relationship between positive symptoms and positive aspects of body image, such as body appreciation and functionality appreciation, in patients with schizophrenia. This study aimed to (1) compare weight stigma, body and functionality appreciation between obese/overweight and normal-weight patients with schizophrenia, and (2) explore the associations between these variables and positive psychotic symptoms in the obese/overweight group.

**Method:**

A cross-sectional study was conducted in the Psychiatric Hospital of the Cross, Lebanon during September 2022 recruiting selected in-patients diagnosed with schizophrenia. Patients were classified as overweight/obese if they had a BMI > 25 (N = 76 (37.25%), aged 55.57 ± 11.30 years, 42.6% females). The Weight self‑stigma questionnaire, the Functionality Appreciation Scale, and the Body Appreciation Scale, and the Positive and Negative Syndrome Scale (PANSS) were used.

**Results:**

No significant difference was found between overweight/obese and normal-weight patients for all variables, except for weight stigma; a significantly higher weight stigma score was significantly found in overweight/obese compared to normal-weight patient. In the bivariate analysis, higher functionality appreciation was significantly associated with higher positive PANSS scores. The results of the linear regression, taking the positive PANSS score as the dependent variable, showed that higher functionality appreciation (Beta = − 0.52) and higher social support (Beta = − 0.16) were significantly associated with lower positive PANSS scores, whereas having a secondary education level compared to illiteracy (Beta = 7.00) was significantly associated with higher positive PANSS scores.

**Conclusion:**

Although based on cross-sectional data, these findings preliminarily suggest that higher functionality appreciation can help reduce the severity of positive psychotic symptoms in overweight/obese schizophrenia patients, and that interventions aimed at improving functionality appreciation could be regarded beneficial therapeutic targets in the treatment of psychosis.

## Background

Schizophrenia is a chronic, complex mental health disorder defined by a wide range of symptoms such as hallucinations, delusions, disordered speech or behavior, and cognitive impairment. Because of its early onset and chronic nature, schizophrenia is very debilitating for many patients [1]. Positive symptoms are the most prominent and recognizable symptoms of schizophrenia. These symptoms can vary in intensity and include hallucinations, delusions, and aberrant motor activity. These core symptoms and concomitant problems associated with schizophrenia may eventually result in social and vocational impairment [2].

Studies revealed that patients diagnosed with schizophrenia are three times more likely to be obese than the general population, with obesity rates reaching up to 60% [3]. The neurobiology of schizophrenia has been related to brain abnormalities that affect energy balance and lead to excessive weight. This raises the possibility that people with schizophrenia may be predisposed to overeating and obesity [4]. Furthermore, the excessive weight gain caused by antipsychotics increases their likelihood of being overweight or obese and has detrimental effects on their general physical health [5]. This weight gain in schizophrenia patients has been associated with a high risk of metabolic syndrome owing to atypical antipsychotic use [5], especially with first-episode psychosis and schizophrenia [6]. Previous studies have also indicated that inflammation in metabolic syndrome plays a significant role in cognitive impairment, leading to poorer cognitive functions in patients with schizophrenia [7]. The monocyte to high-density lipoprotein ratio significantly and positively correlates with body mass index (BMI) and the severity of psychiatric symptoms [8]. Additionally, apelin, vistafin, and resistin, produced by adipocytes, play a role in regulating metabolism and are implicated in the pathophysiology of psychiatric disorders, particularly schizophrenia [9]. Adipokine and other immune compounds have also been found to be associated with metabolic syndrome in patients with schizophrenia [10, 11] Weight gain has also been linked to a range of negative psychological effects in patients, such as a loss of confidence and self-worth, decreased efficacy, a sense of vulnerability, and a self-perceived degraded appearance, which can further impact mood and activity [12]. Overall, it is important to address the issue of obesity in patients with schizophrenia, as it can have significant physical and psychological consequences.

Obesity can exacerbate the challenges faced by individuals with schizophrenia, as it makes them more susceptible to weight stigma. This type of stigma is a societal issue that devalues individuals based on their weight, often leading to preconceived notions that result in prejudice, such as peer rejection, unjust treatment, or explicit discrimination [13, 14]. There is evidence that weight stigma is associated with poor mental health [15], and impaired quality of life among patients with schizophrenia [16]. Following this line of research, we would also expect that weight stigma is associated with more severe positive symptoms; however, we could find no previous studies investigating this relationship.

Apart from obesity, disturbed body experiences have also been reported as highly prevalent in patients with schizophrenia (experienced by 50–70% of patients), and have even been considered as early precursors of the disease [17]. Some of these experiences are related to body image (e.g., attitude to one’s appearance, distortion in body experience [18, 19], rejection of an aspect of the body [20]), or to stimulus perception (e.g., reduced sensation abilities [21–23] underestimated body size perception [24]). Interestingly, these disturbances in body experiences have been shown to be related to positive psychotic symptoms [25–28]. For instance, Sakson-Obada et al. [29] have recently found that positive symptoms were significantly associated with dissatisfaction with appearance, disorders in the sense of body identity, as well as difficulties in regulation of bodily and emotions states; whereas no significant association has been found with negative symptoms. Another study found that [25], who showed that body image pathology was more closely linked to positive symptoms that negative symptoms. Fortunately, social support can help to enhance body-image appreciation [30], which plays an essential role in the improvement of symptoms in patients with schizophrenia [31]. However, while the relationship between negative aspects of body image and positive symptoms was extensively investigated and is relatively well-established, there is a dearth of literature on the relationship between positive symptoms and positive aspects of body image, such as body appreciation and functionality appreciation, in patients with schizophrenia.

Body appreciation is a body construct that refers to holding favorable views toward-, respecting, and accepting one’s body, while appreciating the health and functionality of the body and resisting the internalization of sociocultural appearance standards [32]. Functionality appreciation can be defined as appreciating, respecting, and honoring one’s own body for what it is able to do [33], including physical capacities, bodily senses and sensations, internal processes, self-care, creative endeavors, communication with others [34]. A growing research on body image demonstrated that appreciating the functionality of the body seems to be the most effective method to improve positive body image [35]. According to the objectification theory [36], when body functionality is appreciated, this could enhance positive body image by reducing the tendency to value and evaluate oneself predominantly based on physical appearance [34]. In the same line, when individuals with high BMI appreciate the different domains of body functionality, this may also minimize any overemphasis on physical appearance and body weight [37].

To gain a better understanding of relationship between body-related experiences and positive psychotic symptoms among obese patients with schizophrenia, and to potentially provide new targets for intervention in the areas of prevention and management, this study aimed to : (1) compare weight stigma, body and functionality appreciation between obese/overweight and normal-weight patients with schizophrenia, and (2) to explore the associations between these variables and positive psychotic symptoms in the obese/overweight group. We hypothesized that positive symptoms will be positively associated with weight stigma, and inversely associated with body and functionality appreciation. By identifying these associations, the study hopes to provide new targets for intervention in the areas of prevention and management.

## Methods

### Study design and sampling

**Fig. 1:**
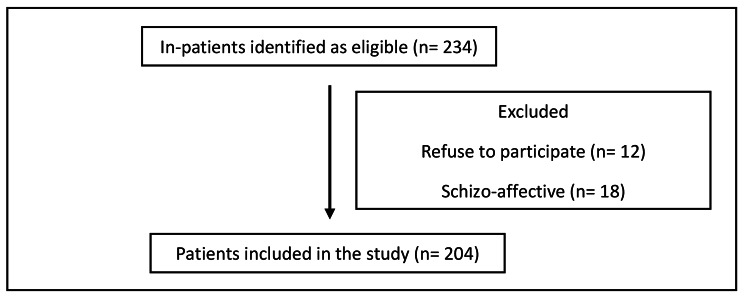
Flow chart of patients included in the study

A cross-sectional study was conducted in September 2022 at the Psychiatric Hospital of the Cross (PHC) recruiting selected in-patients diagnosed with schizophrenia. Patients were to be 18 or older, and diagnosed with schizophrenia based on the Diagnostic and Statistical Manual of Mental Disorders, Fifth Edition criteria [2]. Patients under the age of 18 were excluded, as were those with a major medical condition, cognitive impairments, or a co-diagnosis of one or more mental diseases, such as Alzheimer’s, mental retardation, or epilepsy, as well as those who refused to participate. As a consequence, our sample consisted of 204 individuals (Fig. 1).

### Minimal sample size calculation

We used the G-power to calculate the minimum sample size; the latter was estimated at 75, based on an R^2^ deviation of 0.2 from 0, a 5% alpha risk of error, an 80% power and 10 predictors to be entered in the linear regression model.

### Questionnaire and procedures

The data collection process was done by three trained medical interns. Demographic and clinical information were extracted from the medical file of each patient. Demographic data includes age, marital status, educational level, and duration of illness and hospitalization. Anthropometric measures such as height and weight (to compute the Body Mass Index) were previously measured and written in the patient’s file by the floor nurse. Patients with a BMI > 25 were classified as overweight/obese. The questionnaire was conducted in the Lebanese native language (Arabic) by a face-to-face interview and included the following scales:

### Weight self‑stigma questionnaire (WSSQ)

The 12-item weight self-stigma questionnaire was developed to measure weight self-stigma in overweight and obese people. It helps in evaluating the extent to which overweight or obese individuals dread other people’s opinions of them and the thoughts their minds have been telling them about their weight or body type. The WSSQ contains two distinct subscales, each containing six items: self-devaluation, which involves negative feelings and ideas about being overweight, and fear of enacted stigma, which covers the impression of discrimination and identification with a stigmatized group. Each question is scored on a 5-point Likert scale, with 5 representing full agreement. Higher scores indicate higher levels of self-stigma [38]. The scale has previously been translated and validated in Arabic [39].

### Functionality appreciation scale (FAS)

The Functionality Appreciation Scale is a widely used instrument for the measurement of an individual’s appreciation of their body for what it can do and is capable of doing. It consists of seven items graded on a point scale from strongly disagree (1) to strongly agree (5). Scores have to be summed up; the higher the total score, the greater the appreciation for functionality [33]. The Arabic translation of the scale is already validated in Lebanon [40].

### Body appreciation scale (BAS)

The original Body Appreciation Scale was developed to measure a single dimension of positive body image, which comprises having a positive attitude and accepting one’s physical features. As a result, BAS is regarded as a positive body image measure, with 13 items originally examining individuals’ acceptance, respect, and favorable views about their bodies. The Body Appreciation Scale-2 was refined to include ten items graded on a 5-point Likert scale (1 = never to 5 = always) [32, 41]. The scale is validated in Arabic [42].

### Positive and negative syndrome scale (PANSS)

The Positive and Negative Syndrome Scale (PANSS) assesses the intensity of schizophrenia symptoms. It consists of 30 items divided into three subscales: positive symptoms (7 items), negative symptoms (7 items), and general psychopathology (16 items). Each item is scored on a 7-point Likert scale, with 1 indicating the absence of symptoms and 7 indicating highly severe symptoms. Higher total scores indicate more severe symptoms [43]. The scale has previously been validated in Lebanon [44].

### Multidimensional scale of Perceived Social Support (MDSPSS)

The Multidimensional Scale of Perceived Social Support is a brief, reliable scale used for the assessment of perceived social support. It consists of 12 items divided into three subscales of four items each, covering three dimensions: family, friends, and significant others. Each item is graded on a scale of very strongly disagree (1) to very strongly agree (7) and the results for each item are added together to generate the final score. Higher ratings indicate increased social support [45]. We used the Arabic version, already translated and validated in Lebanon [46].

### Statistical analysis

The data analysis was conducted using the Statistical Package for the Social Sciences (SPSS) software version 26. There was no missing data. All scales and subscales’ reliability were analyzed using Cronbach’s alpha values. When comparing patients who were overweight/obsess to those with a normal BMI in the total sample, the Chi-square test was used to compare two categorical variables, whereas the Student t test was used to compare two means. When selecting overweight/obese patients, the PANSS positive score was normally distributed, with its skewness and kurtosis varying between − 1 and + 1 [47]. The Student t test was used to compare two means, the ANOVA test to compare three or more means and the Pearson test was used to correlate two continuous variables. Effect sizes were calculated using Cramer’s V for categorical variables and using an online software (https://www.socscistatistics.com/effectsize/default3.aspx) for continuous variables; they were categorized as small (d = 0.2), medium (d = 0.5), and large (d = 0.8) [48]. A linear regression was conducted to check for correlates associated with positive PANSS scores. Independent variables entered in the final model were those that showed a p < .25 in the bivariate analysis. P < .05 was deemed statistically significant.

## Results

### Comparison between overweight/obese patients and normal-weight patient

No significant difference was found between overweight/obese and normal-weight patients for all variables, except for weight stigma; a significantly higher weight stigma score was significantly found in overweight/obese compared to normal-weight patient. All details about the sample are summarized in Table 1.

**Table 1:** Comparison between overweight/obese patients and normal-weight patient (n = 204)

Variable	Normal-weight patients (N = 128) (BMI ≤ 25 kg/m^2^)	Overweight/obese patients (N = 76) (BMI > 25 kg/m^2^)	*p*	Effect size
**Sex**			0.260	0.079
Male	89 (65.4%)	47 (34.6%)		
Female	39 (57.4%)	29 (42.6%)		
**Marital status**			0.509	0.046
Single /divorced	16 (57.1%)	12 (42.9%)		
Married	112 (63.6%)	64 (36.4%)		
**Education**			0.379	0.144
Illiterate	51 (66.2%)	26 (33.8%)		
Primary	21 (67.7%)	10 (32.3%)		
Complementary	35 (62.5%)	21 (37.5%)		
Secondary	8 (42.1%)	11 (57.9%)		
University	13 (61.9%)	8 (38.1%)		
Age (in years)	55.13 ± 12.20	55.57 ± 11.30	0.801	0.037
Duration of illness (years)	16.63 ± 11.47	14.11 ± 11.23	0.127	0.222
Duration of hospitalization (years)	14.40 ± 11.62	11.22 ± 10.55	0.052	0.286
Positive PANSS	21.63 ± 9.10	22.00 ± 8.57	0.776	0.042
Body appreciation	42.05 ± 8.08	43.62 ± 6.25	0.578	0.079
Functionality appreciation	18.70 ± 6.48	19.99 ± 5.57	0.149	0.213
Weight stigma	20.55 ± 11.16	23.74 ± 10.91	**0.048**	0.289

Numbers in bold indicate significant *p* values

### Bivariate analysis of factors associated with positive PANSS scores in overweight/obese patients

The bivariate analysis results are displayed in Tables 2 and 3. Higher functionality appreciation was significantly associated with higher positive PANSS scores.

**Table 2:** Bivariate analysis of factors associated with positive PANSS scores in overweight/obese patients (n = 76)

Variable	Mean ± SD	*P*	Effect size
**Sex**		0.806	0.058
Male (n = 47)	21.81 ± 8.83		
Female (n = 29)	22.31 ± 8.29		
**Marital status**		0.856	0.059
Single /divorced (n = 12)	21.58 ± 8.06		
Married (n = 64)	22.08 ± 8.72		
**Education**		0.149	0.313
Illiterate (n = 26)	21.46 ± 8.82		
Primary (n = 10)	23.50 ± 9.59		
Complementary (n = 21)	19.57 ± 6.96		
Secondary (n = 11)	27.45 ± 9.91		
University (n = 8)	20.75 ± 6.45		

Normal weight = BMI ≤ 25 kg/m^2^; Overweight/obese = BMI > 25 kg/m^2^

**Table 3:** Correlation matrix of continuous variables

Variable	1	2	3	4	5	6	7	8	9
1. Positive PANSS	1								
2. Body appreciation	0.05	1							
3. Functionality appreciation	− 0.26*	0.71	1						
4. Social support	− 0.21	− 0.21	− 0.05	1					
5. Weight stigma	0.01	− 0.01	− 0.08	0.06	1				
6. Age	0.07	− 0.13	− 0.31**	− 0.14	− 0.17	1			
7. BMI	− 0.02	0.29*	− 0.09	− 0.04	− 0.07	− 0.28*	1		
8. Duration of illness	0.06	− 0.16	− 0.11	− 0.01	0.09	0.26*	− 0.13	1	
9. Duration of hospitalization	0.11	− 0.11	− 0.21	0.06	0.08	0.40***	− 0.19	0.81***	1

*p < .05; **p < .01; ***p < .001

### Multivariable analysis

The results of the linear regression, taking the positive PANSS score as the dependent variable, showed that higher functionality appreciation (Beta = − 0.52) and higher social support (Beta = − 0.16) were significantly associated with lower positive PANSS scores, whereas having a secondary education level compared to illiteracy (Beta = 7.00) was significantly associated with higher positive PANSS scores (Table 4).

**Table 4:** Linear regression taking the positive PANSS score as the dependent variable

	Unstandardized Beta	Standardized Beta	*P*	95% CI
Functionality appreciation	− 0.52	− 0.34	**0.002**	− 0.85; − 0.19
Social support	− 0.16	− 0.30	**0.007**	− 0.27; − 0.04
Primary education level	− 0.60	− 0.02	0.839	-6.45; 5.25
Complementary education level	-3.45	− 0.18	0.135	-8.02; 1.11
Secondary education level	7.00	0.29	**0.014**	1.47; 12.54
University education level	-3.25	− 0.12	0.314	-9.63; 3.13

ENTER method used in the linear regression; numbers in bold indicate significant p values; Nagelkerke R^2^ = 0.262

## Discussion

We sought to contribute to the literature by investigating the association between positive psychotic symptoms and both negative and positive aspects of body-related experiences in a sample of overweight/obese patients diagnosed with schizophrenia. Results showed that overweight/obese patients had a significantly higher weight stigma score than their normal-weight counterparts. In addition, we showed for the first time that overweight/obese schizophrenia patients with higher appreciation for their functionality reported fewer positive psychotic symptoms. These results have important clinical implications that we discuss later in this paper.

Our results revealed that weight stigma is more prevalent among overweight/obese schizophrenia patients when compared to normal-weight patients. The weight gain and resulting stigma can lead to a loss of self-worth and body changes that amplify a sense of vulnerability. These experiences can have further consequences on mood, activity levels, and psychotic symptoms, such as hearing voices commenting on appearance, which can make weight loss a challenging process [12]. Additionally, the dual stigma associated with obesity and schizophrenia may contribute to the development of mental health problems [49]. Consequently, individuals who are obese face an increased risk of psychological problems, such as depression and anxiety, as well as social problems like isolation due to stigmatization [50]. This stigmatization is also associated with reduced self-esteem and quality of life. Furthermore, people taking antipsychotic medication often view weight gain as one of the most distressing side effects [51]. The negative impact of weight stigma may even extend to psychiatric/mental health medication visits [52]. Interestingly, despite the greater weight stigma experienced by overweight/obese schizophrenia patients, our study found that body appreciation and body functionality do not significantly differ between normal-weight and overweight/obese individuals with schizophrenia. Therefore, the association between all these body-related variables and positive schizophrenia symptoms were addressed in order to better understand their implications for clinical settings to improve the overall mental and physical well-being of individuals with obesity and schizophrenia.

Regarding the association with positive symptoms, it is well-known that body image challenges are widespread among schizophrenia patients, and these have been linked to higher levels of paranoia and auditory hallucinations [53, 54]. In this regard, studies have demonstrated that having concerns about body image may increase the vulnerability of schizophrenia patients, making them feel different, weird, and inferior [55], and thus more likely to experience paranoia [54]. Positive symptoms have been linked to challenges with body identity, increased sensory tolerance, emotional and physical need regulation issues, and a poor self-perception of appearance [56]. Preliminary studies have also suggested that body image concerns can affect the content of voices [53], with schizophrenia patients linking their negative body image to increased paranoid ideation and voices focusing on their physical appearance [57]. Individuals with acute paranoid schizophrenia often exhibit significant body image disorders, and that these symptoms tend to decrease in tandem with the decline of acute psychotic symptoms [24]. Both directly and indirectly, body image concerns may contribute to the persistence of psychotic episodes [53]. However, weight stigma was not shown to be associated to positive symptoms in our sample. Other factors, such as medication adherence or lifestyle behaviors, may have a greater impact on positive symptoms in this population. Further research is needed to understand the complex interplay between weight stigma, positive psychotic symptoms, and other contributing factors.

A major finding of this study is that, while no significant association has been found between body appreciation and psychotic experiences; functionality appreciation remained significantly and negatively associated with positive psychotic symptoms beyond the effect of other covariates included in the linear regression model (education level [56] and social support [58]). These findings provide additional support to the fact that schizophrenia is a disease better characterized by disturbances in terms of body functions than by body image concerns [29]. Our results are in agreement with those of previous studies, which showed that patients with schizophrenia suffered from more abnormal experience of the body (perceiving, interpreting, and regulating body experience) compared to healthy controls; and that these disturbances were significantly related to greater positive symptoms [29]. Overall, this study provides important insights into the complex relationship between body-related experiences and positive psychotic symptoms among obese patients with schizophrenia. Further research is needed to explore these relationships in more detail and to determine the most effective interventions for addressing weight stigma and improving body image among patients with schizophrenia.

### Clinical implications

Despite important advances in pharmacotherapy of schizophrenia-spectrum disorders, a large proportion of patients show treatment-resistance [59], suggesting that novel treatment approaches are highly needed. Our study is the first to demonstrate that a positive aspect of body-related experiences, i.e. functionality appreciation, is negatively associated with positive psychotic symptoms. These findings are in line with prior research suggesting that functionality disturbances appear to be better key features of schizophrenia than body image issues [29]. Although based on cross-sectional data, these findings preliminarily suggest that higher functionality appreciation can help reduce the severity of positive psychotic symptoms in overweight/obese schizophrenia patients, and that interventions aimed at improving functionality appreciation could be regarded beneficial therapeutic targets in the treatment of psychosis. Previous studies demonstrated that functionality-based techniques (e.g., body functionality structured writing exercises) are effective in reducing beliefs that the body is valued positively only through physical appearance, and in promoting a more holistic perception of the body [34, 60]. Such therapeutic approaches need to be tested and adapted to patients with schizophrenia, and could open new avenues for more effective interventions for people with schizophrenia and psychosis.

Given the high rates of obesity and weight gain in schizophrenia patients and the scarcity of research in this area, we call for additional longitudinal research to explore the potential temporal and causal relationships between positive/negative body-related experiences and psychotic symptoms, particularly in the most vulnerable groups of patients with schizophrenia, those who experience unwanted and uncontrollable weight gain triggered by antipsychotics.

### Limitations

The findings of the study are subject to certain limitations. Information bias may be present, as the patients served as the primary source of information, potentially leading to subjective responses or misunderstandings of certain questions. Additionally, selection bias may be present due to the inclusion of patients from a single institution, limiting the generalizability of the findings. Furthermore, the cross-sectional design does not allow for the determination of causality between functionality appreciation and psychotic symptoms. Although superior to the minimum sample size calculated, the sample size is rather small. Despite the fact that all persons responsible for the data collection got a thorough training prior to starting the data collection, interrater variability may be present between the three persons who collected the data. Finally, residual confounding bias may be possible since not all factors linked to psychotic symptoms are taken into consideration.

## Discussion

Our results demonstrate a significant association between high levels of functionality appreciation and decreased psychotic symptoms. This sheds light on the possible role that appreciating the functionality of the body may play in improving positive psychotic symptoms. Further longitudinal research is strongly needed to build on these findings and confirm causality. We also call for future experimental research to test the effects of functionality-based treatment approaches in reducing the severity of psychotic symptoms in schizophrenia patients who present with overweight/obesity and body image concerns.

## Data Availability

All data generated or analyzed during this study are not publicly available to maintain the privacy of the individuals’ identities. The dataset supporting the conclusions is available upon request to the corresponding author.
